# Rapid-acting oral drug (Auvelity) for major depressive disorder

**DOI:** 10.1016/j.amsu.2022.104629

**Published:** 2022-09-13

**Authors:** Yumna Khabir, Mahnoor Rehan Hashmi, Adam Ali Asghar

**Affiliations:** Department of Medicine, Dow University of Health Sciences, Mission Rd, New Labour Colony Nanakwara, Karachi, Sindh, Pakistan

Major depressive disorder (MDD) has been ranked as the third cause of the burden of disease worldwide in 2008 by WHO, which has projected that this disease will rank first by 2030 [1]. The symptoms of MDD include persistently low moods, anhedonia or decreased interest in pleasurable activities, feelings of guilt or worthlessness, lack of energy, poor concentration, changes in appetite, psychomotor retardation, agitation, sleep disturbances, and suicidal thoughts [1]. A diagnosis of MDD requires five of the symptoms above, of which one must be depression or anhedonia causing impairment in social or occupational functioning, as defined by the Diagnostic and Statistical Manual of Mental Disorders, 5th Edition (DSM-5) [1].

It has been discovered that GABA, an inhibitory neurotransmitter, as well as glutamate and glycine, two important excitatory neurotransmitters, are involved in the etiology of depression [1]. GABA levels in the brain, cerebrospinal fluid, and plasma have been reported to be decreased in depressed people [1]. GABA is thought to work as an antidepressant by blocking ascending monoamine pathways, including those in the mesolimbic and mesocortical systems [1].

The common medications recommended for initial treatment of MDD are selective serotonin reuptake inhibitors (Fluoxetine, paroxetine, sertraline, citalopram, and escitalopram), serotonergic noradrenergic reuptake inhibitors (Venlafaxine and duloxetine), bupropion and mirtazapinc^.^ [2] A common problem experienced (60–70% patients) is Treatment-resistant depression (TRD) giving low rate of full recovery [2]. TRD includes suboptimal response to a single antidepressant trial (in terms of dose, duration, and adherence), whereas greater resistance refers to failure of two monotherapy trials or one or more augmentation trials [2]. There has been evidence that the longer it takes to get to fully recovered, the more trials it requires which leads to increased risk of resistance [2]. According to Fava, nearly two-thirds of patients treated with current antidepressants do not respond adequately, and those who do may not experience clinically meaningful results for up to eight weeks [3].

Researchers have looked into medications with *N*-methyl *d*-aspartate (NMDA) receptor antagonist characteristics as potential antidepressants [1]. A first-of-its-kind NMDA receptor antagonist; Auvelity has been approved by the US Food and Drug Administration (FDA) on 18th August 2022 for treating MDD in adults [3]. It is the first and only oral medication with a fast onset of action that has been approved for the treatment of MDD and carries a label indicating statistically significant antidepressant efficacy starting at one week when compared to a placebo [3]. Auvelity combines a N-methyl-d-aspartate (NMDA) receptor antagonist dextromethorphan (45 mg) with a norepinephrine-dopamine reuptake inhibitor bupropion (105 mg) [4]. Since dextromethorphan is quickly metabolized by cytochrome P450 2D6(CYP2D6), it is difficult to achieve therapeutic levels of dextromethorphan using oral route, this is why Bupropion is added along with dextromethorphan since it is a CYP2D6 inhibitor [4]. Auvelity utilizes action mechanisms of several different antidepressant therapies into one [4]. Both dextromethorphan and bupropion increase the availability of norepinephrine by inhibiting its reuptake and also act as alpha-4-beta-2 nicotinic (nACh) antagonists [4]. Bupropion also increases the availability of dopamine by blocking its reuptake [4]. Dextromethorphan acts as NMDA receptor antagonist and increases glutamate levels, it also increases serotonin levels by blocking its reuptake and boosting its action in the dorsal raphe via sigma-1 agonism [3].

According to Maurizio Fava, MD, psychiatrist-in-chief at Massachusetts General Hospital in Boston, the approval of Auvelity represents a milestone in the treatment of depression based on its novel oral NMDA antagonist mechanism, its rapid antidepressant efficacy demonstrated in controlled trials, and a relatively favorable safety profile [1].

Given the crippling nature of depression, the effectiveness of Auvelity as seen at 1 week and maintained subsequently may significantly alter the present paradigm for treating this disorder. The medicine was tested in a thorough clinical program that included more than 1100 people with MDD, according to the manufacturer [3]. The study compared Auvelity to bupropion sustained-release tablets and found Auvelity to be statistically significant in improving depressive symptoms after 6 weeks compared to placebo [3]. Among the common side effects seen after the use of Auvelity were risk of seizures associated with high doses, increased blood pressure (hypertension), manic episodes observed among patients previously diagnosed with bipolar disorder, unusual thoughts or behaviors such as delusions, hallucinations, paranoia and dizziness. Some patients were also diagnosed with eye problems such as angle closure glaucoma and serotonin syndrome after the use of Auvelity [3]. Auvelity is not recommended in pregnancy and breast feeding [3].

More studies investigating the efficacy and efficiency of Auvelity in patients with MDD and other related neurological disorders and comorbidities are required to give a better insight.

## Ethical approval

The approval was not needed.

## Sources of funding for your research

The authors have no financial or proprietary interest in the subject matter of this article.

## Author contribution

Yumna Khabir; Conceptualization, literature review and manuscript writing.

Mahnoor Rehan Hashmi; Literature review and manuscript writing.

Adam Ali Asghar; Literature review and manuscript writing.

## Registration of research studies

Name of the registry:
Unique Identifying number or registration ID:
Hyperlink to your specific registration (must be publicly accessible and will be checked):

## Consent

No consent was required.

## Guarantor

Yumna Khabir, Mahnoor Rehan Hashmi and Adam Ali Asghar.

## Declaration of competing interest

The authors have no conflict of interest.
